# Peptide signaling molecules CLE5 and CLE6 affect Arabidopsis leaf shape downstream of leaf patterning transcription factors and auxin

**DOI:** 10.1002/pld3.103

**Published:** 2018-12-20

**Authors:** Peter DiGennaro, Etienne Grienenberger, Thai Q. Dao, Ji Hyung Jun, Jennifer C. Fletcher

**Affiliations:** ^1^ Plant Gene Expression Center USDA‐ARS/UC Berkeley Albany California; ^2^ Department of Plant and Microbial Biology University of California Berkeley California; ^3^ Present address: Department of Entomology and Nematology University of Florida Gainesville Florida; ^4^ Present address: Centre National de la Recherche Scientifique (CNRS) Institute of Plant Molecular Biology University of Strasbourg Strasbourg France; ^5^ Present address: BioDiscovery Institute and Department of Biological Sciences University of North Texas Denton Texas

**Keywords:** Arabidopsis, CLE, leaf development, signaling, WOX

## Abstract

Intercellular signaling mediated by small peptides is critical to coordinate organ formation in animals, but whether extracellular polypeptides play similar roles in plants is unknown. Here we describe a role in Arabidopsis leaf development for two members of the CLAVATA3/ESR‐RELATED peptide family, CLE5 and CLE6, which lie adjacent to each other on chromosome 2. Uniquely among the *CLE* genes, *CLE5* and *CLE6* are expressed specifically at the base of developing leaves and floral organs, adjacent to the boundary with the shoot apical meristem. During vegetative development *CLE5* and *CLE6* transcription is regulated by the leaf patterning transcription factors BLADE‐ON‐PETIOLE1 (BOP1) and ASYMMETRIC LEAVES2 (AS2), as well as by the WUSCHEL‐RELATED HOMEOBOX (WOX) transcription factors WOX1 and PRESSED FLOWER (PRS). Moreover, *CLE5* and *CLE6* transcript levels are differentially regulated in various genetic backgrounds by the phytohormone auxin. Analysis of loss‐of‐function mutations generated by genome engineering reveals that *CLE5* and *CLE6* independently and together have subtle effects on rosette leaf shape. Our study indicates that the CLE5 and CLE6 peptides function downstream of leaf patterning factors and phytohormones to modulate the final leaf morphology.

## 1 INTRODUCTION

Plants are unique in their ability to generate new organs and tissues throughout their life span, producing intricate structures such as flowers and leaves via complex molecular regulatory mechanisms (Bar & Ori, 2014; Tsukaya, 2013). During vegetative development, leaves initiate as small, regularly spaced primordia on the flanks of the shoot apical meristem. Following leaf initiation, individual primordia develop along three axes of polarity: the adaxial‐abaxial, proximal‐distal, and medial‐lateral axes. Early polarization along these three axes serves to specify the unique cell types within the emergent leaf. In simple‐leaved species, such as Arabidopsis, subsequent cell growth and differentiation then results in a mature three‐dimensional structure with a narrow petiole and a broad lamina, or blade (Kalve, De Vos, & Beemster, 2014). The blade tissue contains an epidermal layer of jigsaw‐shaped pavement cells bounded by several narrow layers of elongated cells at the margin, and is specialized for light capture. Yet despite the importance of leaves as the main sites for photosynthesis, as well as carbon fixation and gas exchange in plants (Tsukaya, 2013), much remains to be understood about the genetic mechanisms that control leaf formation, shape, and function.

The coordination of complex developmental activities such as leaf formation by growing plants is critically dependent on the communication of information between cells. Long‐range intercellular signaling is mediated by phytohormones such as cytokinin, auxin, gibberellin (GA), abscisic acid (ABA), and brassinosteroids (BL). Among these hormones, auxin, GA and BL have well‐characterized roles in orchestrating leaf development (Kalve et al., 2014). In addition to setting the positions of newly arising leaf primordia, auxin contributes to the establishment of leaf adaxial‐abaxial polarity as well as the coordinated transition from cell proliferation to cell expansion. GA and also BL regulate cell division and expansion during the leaf maturation process. These phytohormone‐mediated effects on leaf morphogenesis can occur via changes in hormone biosynthesis, as in the case of GA and BL, and/or in hormone transport or response, as in the case of auxin (Kalve et al., 2014; Tsukaya, 2013).

In addition to phytohormone signaling pathways, families of secreted signaling peptides are involved in regulating plant developmental events (Grienenberger & Fletcher, 2015; Matsubayashi, 2014). The CLAVATA3/EMBRYO SURROUNDING REGION‐related (CLE) family is one of the largest and best‐studied secreted signaling peptide families in plants. CLE family members are found throughout the plant kingdom as well as in some plant‐parasitic nematodes (Miyawaki, Tabata, & Sawa, 2013). The Arabidopsis genome encodes 32 *CLE* gene family members, which are expressed in a wide variety of tissues and developmental stages (Jun, Fiume, et al., 2010). The genes encode secreted 12‐ to 13‐amino‐acid mature polypeptides, derived from a conserved C‐terminal CLE domain, which undergo various posttranslational modifications (Matsubayashi, 2014).

Although intercellular signaling molecules are crucial for orchestrating plant growth and development, determining their biological activities has proven challenging. To date only a handful of Arabidopsis CLE family members have defined functions. The founding CLE family member CLAVATA3 (CLV3) acts in a negative feedback loop that regulates stem cell homeostasis in the shoot apical meristem (Brand, Fletcher, Hobe, Meyerowitz, & Simon, 2000; Schoof et al., 2000). In addition CLE40 plays a role in root stem cell homeostasis, and CLE41 and CLE44 function in vascular development and lateral root formation (Matsubayashi, 2014; Wang, Zhang, & Wu, 2016). Although a comprehensive library of *CLE* loss‐of‐function alleles now exists (Yamaguchi et al., 2017), plants carrying most single *cle* null alleles show no obvious developmental or physiological phenotypes (Jun, Fiume, et al., 2010). One possible explanation is that due to a high degree of sequence homology, many *CLE* genes play largely redundant roles in plant biology (Strabala et al., 2006).

Key components of CLE‐mediated signaling pathways are members of the WUSCHEL‐RELATED HOMEOBOX (WOX) family of homeodomain‐containing transcription factors (Haecker et al., 2004). Expression of the founding Arabidopsis *WOX* gene family member, *WUSCHEL (WUS)*, is limited by CLV3 signaling to the most central region of the shoot apical meristem (Laux, Mayer, Berger, & Jurgens, 1996), where it functions in a non‐cell‐autonomous manner to maintain stem cell activity in the overlying cells (Brand, Grunewald, Hobe, & Simon, 2002; Schoof et al., 2000; Yadav et al., 2011). In roots, CLE40 likewise restricts the expression domain of *WOX5* (Stahl, Wink, Ingram, & Simon, 2009), which acts non‐cell‐autonomously to promote columella stem cell maintenance (Sarkar et al., 2007). The identical CLE41 and CLE44 peptides induce *WOX4* and *WOX14* expression to promote vascular cell division (Etchells, Provost, Mishra, & Turner, 2013; Hirakawa, Kondo, & Fukuda, 2010). These examples suggest that the use of CLE‐WOX signaling modules during plant development is widespread.

Evidence is accumulating that CLE polypeptide signaling pathways intersect with classical phytohormone signaling pathways to direct various plant developmental processes (Wang et al., 2016). For example, the CLV3 pathway target WUS maintains shoot and floral meristem activity by directly regulating components of cytokinin response pathways (Leibfried et al., 2005). CLE40 controls the expression of auxin, cytokinin, and ABA signaling genes to inhibit cell differentiation in the root apical meristem (Pallakies & Simon, 2014). CLE6 and CLE41/44 peptide‐stimulated vascular cell proliferation is positively regulated by auxin, and exogenous CLE6 peptide application induces the expression of auxin signaling‐related promoters such as *proPIN1*:GUS in the hypocotyl stele (Whitford, Fernandez, De Groodt, Ortega, & Hilson, 2008). In addition, GA promotes *CLE6* expression in the root stele, and *CLE6* over‐expression can partly suppress the phenotypes of GA‐deficient plants (Bidadi et al., 2014). Based on such observations it has been proposed that CLE peptides may play general roles in controlling stem cell fate via their communication with plant hormone‐regulated signaling networks (Whitford et al., 2008).

Yet although there is increasing evidence that CLE peptide and phytohormone signaling pathways connect to regulate Arabidopsis growth and development, most studies to date have been conducted using root or vascular tissues (Wang et al., 2016). In contrast, beyond the role of *CLV3* in shoot apical meristem maintenance, very little is known about the regulation or activity of *CLE* genes in shoot or shoot‐derived tissues. However, a good deal of work has focused on the regulation of leaf development, including the adaxial side adjacent to the SAM boundary as well as at the base of the developing floral organs (Ha, Jun, Nam, & Fletcher, 2004; Hepworth, Zhang, McKim, Li, & Haughn, 2005; Norberg, Holmlund, & Nilsson, 2005). Two closely related genes that encode transcriptional regulatory proteins, *BLADE‐ON‐PETIOLE1* (*BOP1)* and *BOP2,* are expressed at the base of developing rosette leaves and have largely redundant functions in developing lateral organs, including suppressing ectopic blade outgrowth from the leaf petiole (Ha, Jun, Nam, & Fletcher, 2007; Ha et al., 2003). The BOP proteins activate the transcription of *ASYMMETRIC LEAVES2 (AS2)* in the proximal region of developing leaf primordia. *AS2* encodes a member of the LATERAL ORGAN BOUNDARIES (LOB) family of leucine‐zipper proteins (Iwakawa et al., 2002; Lin, Shuai, & Springer, 2003; Xu et al., 2003) that physically interacts with the ARP domain transcription factor AS1 (Xu et al., 2003) to promote lateral organ identity and adaxial leaf polarity (Machida, Nakagawa, Kojima, Takahashi, & Machida, 2015). *AS2* transcription occurs in developing leaf and floral primordia in a broad domain (Byrne et al., 2000; Iwakawa et al., 2007) that overlaps with those of *BOP1* and *BOP2*.

Here we investigate the expression and function of the closely related *CLE5* and *CLE6* genes, which lie adjacent to one another on chromosome 2 and encode identical CLE polypeptides (Cock & McCormick, 2001). We show that *CLE5* and *CLE6* have highly specific, overlapping expression patterns at the base of lateral organ primordia. We find that, despite having a high degree of overall sequence similarity, they are differentially regulated during vegetative development by the *BOP* and *AS2* genes, the WOX transcription factors PRS and WOX1, and by auxin. Using null alleles of *CLE5* and *CLE6* generated by genome editing, we demonstrate that although neither single nor double mutations in *CLE5* and *CLE6* have a detectable impact on Arabidopsis organ initiation or patterning, they have subtle effects on overall leaf shape. Our studies indicate that *CLE5* and *CLE6* act downstream of leaf patterning factors and phytohormones to direct formation of the final leaf morphology.

## 2 EXPERIMENTAL PROCEDURES

### 2.1 Plant material and growth conditions

Seeds were imbibed at 4°C for 5 days before sowing and plants were grown in Percival growth chambers at 21°C under long day conditions (16 hr light, 8 hr dark) with a light fluence rate of approximately 110 μmol m^−2^ s^−1^. Transgenic plants carrying the pCLE5:*GUS* or pCLE6:*GUS* constructs were generated as described (Jun, Fiume, et al., 2010), using 2,570 bp upstream of the *CLE5* translation start site or 1,713 bp upstream of the *CLE6* translation start site. Promoter alignments were performed using the EMBOSS Needle program for pairwise sequence alignment (Rice, Longden, & Bleasby, 2000). For the generation of the pBOP1:*CLE6 bop1‐4 bop2‐11* lines, a 5797 bp *BOP1* promoter fragment was PCR‐amplified using the primers pBOP1(‐5797) FW and pBOP1 RV (primers listed in Supporting Information Table S1). The *CLE6* coding sequence was PCR‐amplified using the primers pBOP1:CLE6 FW and CLE6_CDS+Stop RV (Supporting Information Table S1). Both PCR products were gel‐purified and used as a template for a third PCR with the primers pBOP1(‐5797) FW and CLE6_CDS+Stop RV to generate a pBOP1:*CLE6* product. The product was subcloned into the TOPO pCR8‐GW vector (ThermoFisher) and recombined using Gateway technology into the pEarley Gate 300 destination vector. The recombined vector was used for floral dip transformation with Agrobacterium tumefasciens GV3101. T1 transgenic plants were analyzed for *CLE6* expression. All lines were in the Arabidopsis Columbia‐0 (Col‐0) background unless otherwise stated.

### 2.2 Histochemical assays

GUS staining of 10 to 14‐day‐old seedlings was performed as described (Jun, Fiume, et al., 2010). For Dex treatments, seedlings were transferred into half strength liquid Murashige and Skoog (MS) media and then either mock‐treated with 70% ethanol (as a solvent for Dex) or treated with Dex at a final concentration of 10 μM for 4 hr. RNA in situ hybridization was performed on 7‐day‐old seedlings as described (Jun, Ha, & Fletcher, 2010). Non‐radioactively labeled probes were generated from the full cDNA sequences of *CLE5* and *CLE6* transcripts using primers listed in Supporting Information Table S1.

### 2.3 p35S:*BOP1‐GR* and p35S:*AS2‐GR* Activation Assays

Eleven‐day‐old p35S:*BOP1‐GR bop1‐1* or p35S:*AS2‐GR as2‐1* seedlings were transferred into half strength liquid MS media. Seedlings were then mock‐treated with 70% ethanol or treated with Dex at a final concentration of 10 μM, incubated under slight agitation, and harvested between 30 min and 4 hr for RT‐qPCR analysis. For the hormone assays, a final concentration of 10 μM IAA, 10 μM NAA, 20–30 μM GA_4_, or 2 μM BL was added to the wild‐type and p35S:*BOP1‐GR bop1‐1* seedlings at the same time as the Dex.

### 2.4 RT‐qPCR analyses

For RT‐qPCR studies, total RNA was extracted using TriReagent (Sigma‐Aldrich), treated with DNase I and purified with the RNeasy Mini Kit (Qiagen). Four to five μg of total RNA were used for reverse transcription with SuperScript III and oligo(dT_15_) (ThermoFisher Scientific). Quantitative real‐time Polymerase Chain Reaction (PCR) was performed using the iTaq Universal Sybr Green Supermix (Bio‐Rad) with the primers listed in Supporting Information Table S1. PCR reactions were run and analyzed using a MyiQTM Single‐Color real‐time PCR detection system (Bio‐Rad). Two‐step PCR conditions were as follows: initial denaturation at 95°C for 3 min, followed by 40 cycles of 95°C for 10 s and 60°C for 30 s. Quantification of relative gene expression was performed using the ΔΔCt method (Livak & Schmittgen, 2001), and calculated based on at least three biological replicates with three technical replicates each. Expression values were normalized to *TUBULIN2 (TUB2)* or *MON1* (Czechowski, Stitt, Altmann, Udvardi, & Scheible, 2005).

### 2.5 ChIP‐qPCR analyses

ChIP was performed as described (Yamaguchi et al., 2014) using an anti‐GR antibody (SC‐1004, Santa Cruz Biotechnology). Seedlings were mock‐treated in 70% ethanol or Dex‐treated for 4 hr. Quantification of immunoprecipitated DNA was performed by semi‐quantitative PCR using the primers listed in Supporting Information Table S1.

### 2.6 CRISPR/Cas9 cloning and analysis

The Cas9 and sgRNA cassettes were PCR‐amplified from the At‐psgR‐Cas9 vectors (obtained from Dr. Jian‐Kang Zhu, Purdue University) using M13 FW and M13 RV primers and subcloned into the pCR8/GW/TOPO vector (ThermoFisher Scientific) to add the attL1 and attL2 Gateway sequences. The CRISPR/Cas9 Gateway‐compatible cassette was then PCR‐amplified using primers T7_promoter and pCR8_FW (Supporting Information Table S1) and cloned into the GEM‐T vector (Promega) for subsequent experiments (named thereafter At‐psgR/GW). The genomic target sequences for *CLE5* and *CLE6* were 5′‐AGTTCCGACAGGGTTTCACCCGG‐3′ and 5′‐TACATATCGCCCCACAACCATGG‐3′, respectively. The At‐psgR/GW plasmid was digested with *BbsI* restriction enzyme and used for ligation with the annealed primers CLE5 P1 and P2 or CLE6 P1 and P2 (Supporting Information Table S1). At‐psgR/GW plasmids containing the *CLE5* or *CLE6* genomic target sequences were transferred into the pEarleyGate 301 vector using the LR enzyme mix (ThermoFisher Scientific). The recombinant pEarleyGate 301 constructs were transferred into *Agrobacterium tumefaciens* GV3101 and transformed into Arabidopsis Col‐0 using the floral dip method (Clough & Bent, 1998).

Genotyping the *CLE5* CRISPR alleles was performed by using the primers CLE5CR_FW and CLE5CR_RV (Supporting Information Table S1) in a PCR reaction to amplify a 727 base pair (bp) product. Digesting the PCR product with *HphI* yielded 384 bp and 343 bp bands from wild‐type tissue, whereas the product from mutant tissue remained undigested. Genotyping the *CLE6* CRISPR alleles was performed by using the primers CLE6CR_FW and CLE6CR_RV (Supporting Information Table S1) in a PCR reaction to amplify an 882 bp product. Digesting the PCR product with *MslI* yielded 545 bp and 337 bp bands from wild‐type tissue, whereas the product from mutant tissue remained undigested. T2 mutant plants lacking the Cas9 cassette were identified by PCR using primers sgRNA_FW and sgRNA_RV specific to the Cas9 sequence.

### 2.7 Phenotypic analysis

Leaf morphometrics experiments were conducted using LeafAnalyser software as described (Weight, Parnham, & Waites, 2008), using 50 landmarks per leaf sample and >50 leaf samples per genotype. Principal component analyses were also conducted using LeafAnalyser by calculating eigenvectors (principal components) and eigenvalues (variances) from the covariance matrix generated by LeafAnalyser of each landmark from each leaf (see Weight et al., 2008; Supporting Information Appendix S2). Histological analysis was performed as described (Ha et al., 2003). Scanning electron microscopy was performed as described (Fiume, Pires, Kim, & Fletcher, 2010) and samples visualized on a Hitachi S4700 scanning electron microscope.

## 3 RESULTS

### 3.1 *CLE5* and *CLE6* have distinct yet overlapping expression patterns

**Figure 1:**
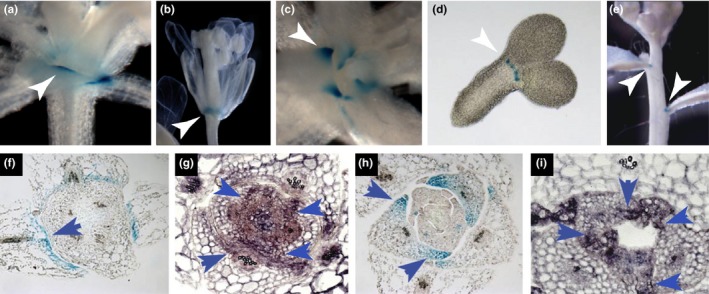
*CLE5* and *CLE6* expression in wild‐type Arabidopsis plants. (a–b) pCLE6:*GUS* promoter activity at the base of wild‐type Col‐0 (a) rosette leaves and (b) floral organs. (c–e) pCLE5:*GUS* promoter activity at the base of Col‐0 (c) rosette leaves, (d) embryo cotyledons, and (e) cauline leaves. (f–g) *CLE6* mRNA expression in transverse sections of leaves from 7‐day‐old wild‐type plants. (h–i) *CLE5* mRNA expression in transverse sections of leaves from 7‐day‐old wild‐type plants. Arrowheads indicate gene expression at the base of the organ

To investigate *CLE5* and *CLE6* transcription patterns in detail throughout the Arabidopsis life cycle, we performed promoter:GUS and in situ hybridization analysis of the two genes in aerial tissues. In wild‐type Col‐0 plants pCLE6:*GUS* specific promoter activity was detected at the base of young rosette leaves (Figure 1a) and at the base of the floral organs (Figure 1b). Similarly, specific pCLE5:*GUS* activity was detected at the base of young rosette leaves (Figure 1c), at the base of the cotyledons in mature embryos (Figure 1d), and at the base of the cauline leaves (Figure 1e). pCLE6:*GUS* promoter activity was also found at the base of the cotyledons. Previously reported pCLE6:*GUS* expression included the base of the cauline leaves at the primary branching point on the inflorescence stem and pCLE5:*GUS* expression in floral organs (Jun, Fiume, et al., 2010). Overall, activity of the *CLE5* and *CLE6* promoters was restricted to the most proximal region of lateral organ primordia, adjacent to the boundary with the shoot meristem.

Transverse sections through wild‐type vegetative shoot apices revealed further region‐specific expression of *CLE5* and *CLE6*. pCLE6:*GUS* promoter activity in the developing rosette leaves was confined to the adaxial domain (Figure 1f), and this expression pattern was confirmed using RNA in situ hybridization (Figure 1g). In contrast, *CLE5* expression occurred in both the adaxial and abaxial domains (Figure 1h,i). Furthermore, *CLE5* and *CLE6* displayed reciprocal expression patterns along the medio‐lateral axis of developing rosette leaf primordia. *CLE6* was transcribed predominantly within the medial domain above the midvein (Figure 1a,f,g) whereas *CLE5* transcription was restricted to the lateral domain at the very edges of the primordia (Figure 1c,h,i). Thus, although the promoter activity patterns of *CLE5* and *CLE6* overlap extensively, the two genes display distinct expression patterns along the adaxial‐abaxial and medial‐lateral polarity axes within the developing leaf primordia.

### 3.2 Known leaf patterning transcriptional factors regulate *CLE5* and *CLE6* expression

**Figure 2:**
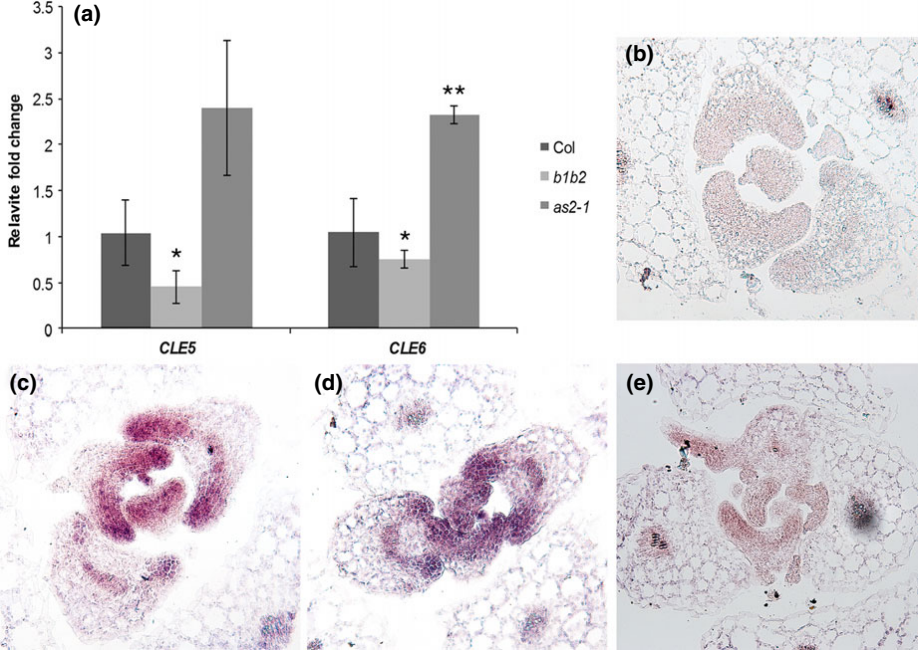
*CLE5* and *CLE6* expression in leaf patterning mutants. (a) Relative fold change in *CLE5* and *CLE6* transcript levels in 10‐day‐old Col‐0, *bop1‐4 bop2‐11 (b1b2)*, and *as2‐1* seedlings. Expression values (mean ± *SD*) were normalized to *MON1* and asterisks indicate a significant difference from the wild‐type mean (*p < 0.05; **p < 0.01) using Student's *t* test. (b) Transverse section of a Col‐0 seedling hybridized with a *CLE6* sense probe. (c) Transverse section of a Col‐0 seedling hybridized with a *CLE6* antisense probe. (d) Transverse section of a *b1b2* seedling hybridized with a *CLE6* antisense probe. (e) Transverse section of an *as2‐1* seedling hybridized with a *CLE6* antisense probe

The *CLE5* and *CLE6* expression patterns at the base of developing lateral organs were very similar to those reported for the *BOP1* and *BOP2* leaf patterning genes (Ha et al., 2004; Hepworth et al., 2005; Norberg et al., 2005), as well as for their downstream target *AS2* (Byrne et al., 2000; Iwakawa et al., 2007). Given the overlap in expression patterns between these genes, we examined whether *CLE5* and/or *CLE6* transcription was regulated by *BOP* or *AS2* gene activity in developing rosette leaf primordia using RT‐qPCR. Compared to wild‐type 10‐day‐old Col‐0 seedlings, *CLE5* and *CLE6* expression was reduced by 25%–50% in *bop1‐4 bop2‐11* (*b1b2*) null mutant seedlings (Figure 2a), indicating that BOP1 and BOP2 are positive regulators of *CLE5* and *CLE6* transcription. Conversely, in *as2‐1* seedlings, *CLE5* and *CLE6* expression levels were elevated compared to wild‐type (Figure 2a), indicating that AS2 negatively regulates *CLE5* and *CLE6* transcription. Taken together, these results indicate that *CLE5* and *CLE6* function downstream of BOP1/2 and AS2 transcriptional regulation as direct or indirect targets of these key leaf patterning factors.

The BOP1/2 and AS2 proteins could regulate *CLE5* and *CLE6* transcription by affecting their mRNA levels, their expression domains within developing leaves, or both. To determine which, we performed in situ hybridization experiments using 10‐day‐old Col‐0, *b1b2,* and *as2‐1* seedling tissues. Compared to the sense probe, which showed no specific *CLE6* expression in wild‐type Col‐0 leaves (Figure 2b), the antisense probe detected strong *CLE6* expression across the adaxial domain of young Col‐0 leaf primordia and the marginal region (tips) of older primordia (Figure 2c). This expression pattern was unchanged in *b1b2* leaf primordia (Figure 2d), indicating that BOP1 and BOP2 induce *CLE6* mRNA expression levels without altering its expression domain. Similarly, we detected no difference in the *CLE6* expression domain between developing Col‐0 and *as2‐1* leaves (Figure 2e), showing that the elevation in *CLE6* mRNA levels in *as2* leaves does not result from an enlarged expression domain. These data indicate that neither BOP1/2 nor AS2 affect the *CLE6* expression pattern but that a combination of activators and repressors is required to restrict *CLE5* and *CLE6* transcription to the appropriate levels in developing rosette leaves.

**Figure 3:**
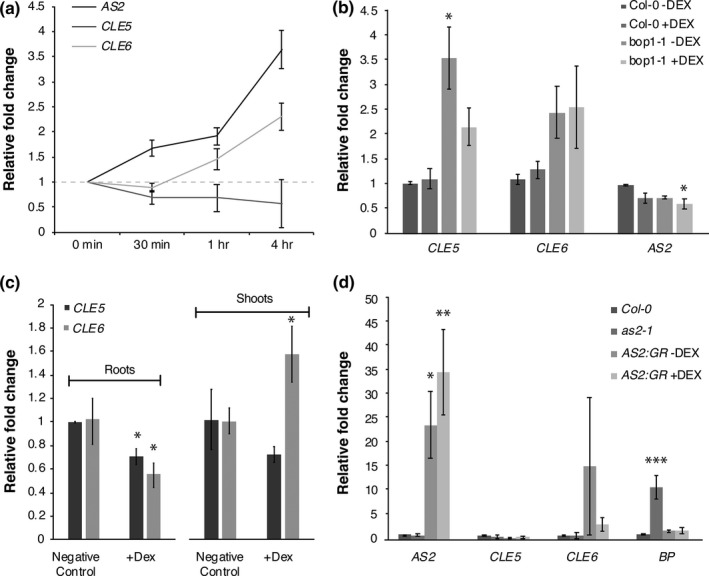
*CLE5* and *CLE6* expression in response to BOP1 or AS2 induction. (a) Time course of *AS2*,*CLE5* and *CLE6* transcript levels in p35S:*BOP1‐GR bop1‐1* seedlings treated with Dex for 0 to 4 hr. (b) Relative fold change in *AS2, CLE5* and *CLE6* transcript levels in Col‐0 and *bop1‐1* seedlings treated with Dex for 4 hr. (c) Relative fold change in *CLE5* and *CLE6* transcript levels in root or shoot tissues from p35S:*BOP1‐GR bop1‐1* seedlings treated with Dex for 4 hr. (d) Relative fold change in *AS2, CLE5, CLE6* and *BP* transcript levels in shoot tissues from *as2‐1* and p35S:*AS2‐GR as2‐1* seedlings treated with Dex for 2 or 4 hr. Expression values (mean ± *SD*) were normalized to *TUB2* or *MON1* and asterisks indicate a significant difference from the wild‐type mean (*p < 0.05; **p < 0.01; ***p < 0.001)

Next, we investigated whether the regulation of the two *CLE* genes by BOP1 was direct or indirect by conducting a time course of *CLE5* and *CLE6* transcription in p35S:*BOP1‐GR bop1‐1* transgenic plants. In this system, application of the hormone dexamethasone (Dex) translocates the ectopically produced BOP1 transcriptional regulatory protein into the nucleus, rescuing the dominant negative *bop1‐1* ectopic leaf outgrowth phenotype (Jun, Ha, et al., 2010) that phenocopies the *b1b2* null mutant phenotype (Ha et al., 2004). We first examined *AS2* transcription over the 4‐hr time course as a positive control. Consistent with previous data (Jun, Ha, et al., 2010), we found that *AS2* transcription was induced by BOP1‐GR after 30 min of 10 μM Dex application and continued to be induced over the full 4 hr of Dex treatment (Figure 3a). *CLE6* transcription was significantly upregulated by BOP1 after 1 hr of Dex treatment, and its expression levels continued to increase to >2 fold after 4 hr (Figure 3a). Thus BOP1 is sufficient to induce *CLE6* transcription, although only to moderate levels. The rapid activation of *CLE6* transcription after 1 hr of Dex application suggests that *CLE6* could be an immediate target of BOP1 induction. In contrast, *CLE5* expression was slightly downregulated after 30 min of Dex treatment and remained steady thereafter (Figure 3a), indicating that BOP1 is insufficient to induce *CLE5* transcription on its own.

As a control for the Dex treatment itself we applied 10 μM Dex for 4 hr to Col‐0 and *bop1‐1* plants and quantified *AS2, CLE5,* and *CLE6* mRNA levels. We found that Dex application to wild‐type plants had no effect on *AS2, CLE5,* or *CLE6* transcript abundance (Figure 3b), although we observed considerable variability in the transcript levels within each genotype because only a very small number of cells within the total leaf tissue assayed express *CLE5* or *CLE6*. Dex application to *bop1‐1* plants lacking the p35S:*BOP1‐GR* transgene also did not affect *AS2* or *CLE6* mRNA levels; however, *CLE5* mRNA levels were reduced by approximately 40% compared to mock‐treated *bop1‐1* plants. Thus the slight reduction in *CLE5* mRNA levels during the time course can be attributed to a repressive effect of Dex application to *bop1‐1* plants rather than of BOP1‐GR activity.

Both *CLE5* and *CLE6* are expressed in root tissues as well as in shoot tissues (Jun, Fiume, et al., 2010), so we determined whether the effect of BOP1 on *CLE5* and/or *CLE6* expression was limited to one of the two tissue types. We treated p35S:*BOP1‐GR bop1‐1* plants with 10 μM Dex for 4 hr, isolated shoot and root tissues, and measured *CLE5* and *CLE6* mRNA levels using RT‐qPCR. We found that although *CLE5* expression levels were unaltered in shoot versus root tissues, BOP1 induction of *CLE6* expression occurred in the shoot tissues but not in the root tissues (Figure 3c). Thus, the regulation of *CLE6* transcription by BOP1 is restricted to above‐ground shoot tissues that contain the leaf primordia.

To determine if *CLE6* induction by BOP1 was due to direct transcriptional regulation, we tested whether BOP1 protein directly associated with *CLE6* regulatory sequences. We performed chromatin immunoprecipitation (ChIP‐qPCR) assays with Dex‐treated p35S:*BOP1‐GR bop1‐1* seedlings using primer sets spanning 3.2 kilobases (kb) upstream of the *CLE5* coding region, the intergenic region between *CLE5* and *CLE6,* and 1 kb downstream of the *CLE6* coding region. No BOP1 binding to these *CLE5* and *CLE6* regulatory regions was detected (Supporting Information Figure S1), although BOP1 binding was detected as expected to regulatory sites within the *AS2* promoter (Jun, Ha, et al., 2010). Therefore the regulation of *CLE6* by BOP1 appears to occur indirectly through an intermediary factor. We also attempted to rescue the *bop1‐4 bop2‐11* ectopic blade outgrowth phenotype (Ha et al., 2007) by directing *CLE6* expression within the BOP domain under the control of 6.0 kb of *BOP1* promoter sequence. This promoter region is sufficient to drive *BOP1* transcription in its native domain (Jun, Ha, et al., 2010). However, none of the pBOP1:*CLE6 bop1‐4 bop2‐11* lines exhibited rescue of the ectopic blade outgrowth phenotype, indicating that *CLE6* expression in the *BOP1* domain alone is not sufficient to restore wild‐type petiole identity. Thus it is likely that rescue of the *bop* phenotype requires *CLE6* expression beyond the *BOP* expression domain and/or other factors in addition to *CLE6*.

The observation that BOP1 was not a direct regulator of *CLE5* or *CLE6* transcription suggested that role would fall to a downstream component of the BOP1 regulatory pathway. Our time course showed that *AS2* induction by BOP1‐GR occurred prior to *CLE6* induction, so we tested whether the AS2 transcription factor might directly regulate *CLE5* and/or *CLE6* transcription. We analyzed *CLE5* and *CLE6* mRNA levels in shoots of 11‐day‐old p35S:*AS2‐GR as2‐1* seedlings after 2 and 4 hr of Dex induction using RT‐qPCR. At both time points we observed a slight decrease in *CLE5* mRNA levels compared to mock‐treated seedlings (Figure 3d); however, this decline was not statistically significant. We also detected no significant change in *CLE6* mRNA levels at either time point (Figure 3d). These data show that AS2 alone is not sufficient to affect *CLE5* and/or *CLE6* transcription, either because the *CLE* genes are not direct AS2 regulatory targets or because the amount of its partner protein AS1 becomes rate‐limiting when AS2 is over‐expressed.

**Figure 4:**
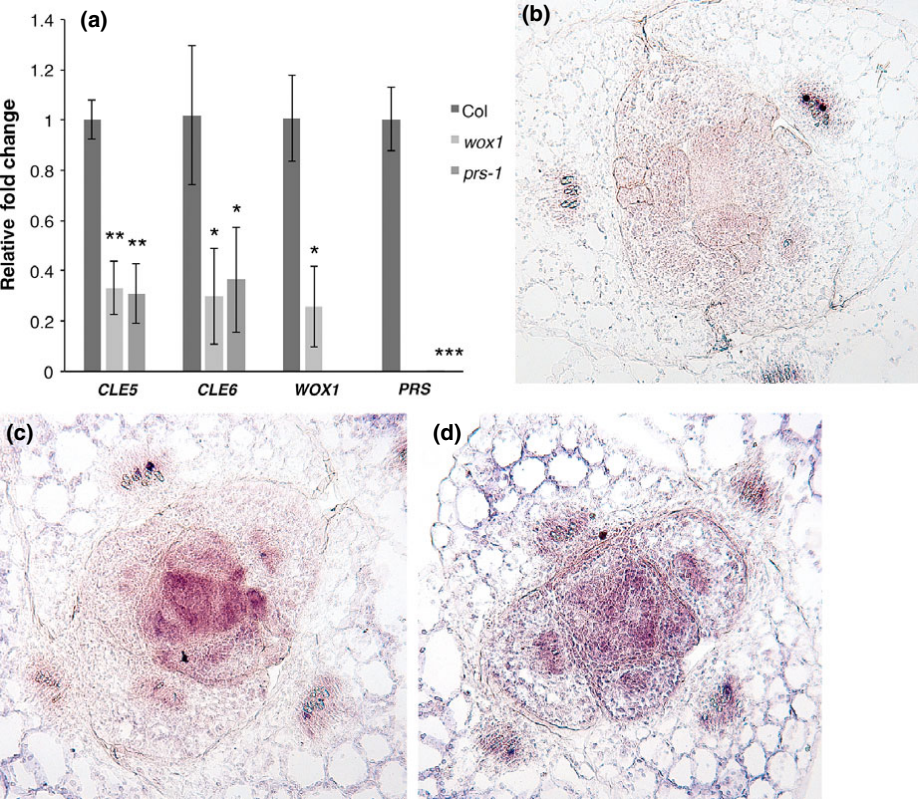
*CLE5* and *CLE6* expression in *prs* and *wox1* mutants. (a) *CLE5* and *CLE6* transcript levels in 10‐day‐old Col‐0 and *prs‐1* and *wox1‐1* seedlings. Expression values (mean ± *SD*) were normalized to *MON1* and asterisks indicate a significant difference from the wild‐type mean (*p < 0.05; **p < 0.01; ***p < 0.001). (b) Transverse section of a Col‐0 seedling hybridized with a *CLE6* sense probe. (c) Transverse section of a Col‐0 seedling hybridized with a *CLE6* antisense probe. (d) Transverse section of a *prs‐1 wox1‐1* seedling hybridized with a *CLE6* antisense probe

Other well‐characterized players in *CLE* gene regulation are members of the WOX family of transcription factors. Among these, *WOX1* and *PRESSED FLOWER (PRS)*, also known as *WOX3*, have described roles in early leaf development (Nakata et al., 2012), and act in blade outgrowth and leaf margin formation. To assess whether these WOX transcription factors regulated *CLE5* and *CLE6* transcription, we measured *CLE5* and *CLE6* mRNA levels in the shoots of 10‐day‐old *wox1‐1* and *prs‐1* null mutant seedlings. Expression of *CLE5* and *CLE6* was reduced by about 50% in both *prs* and *wox1* seedlings compared to wild‐type Col‐0 (Figure 4a), indicating that WOX1 and PRS are each positive regulators of *CLE5* and *CLE6* transcription. In situ hybridization showed no alteration in the *CLE6* expression domain in 10‐day‐old *prs wox1* seedlings compared to Col‐0 (Figure 4c,d), indicating that PRS and WOX1 do not affect the *CLE6* spatial domain. We also noted that *WOX1* transcripts were absent from *prs‐1* seedlings and *PRS* transcripts were absent from *wox‐1* seedlings (Figure 4a). Thus *PRS* and *WOX1* are required for one another's expression in developing leaf primordia.

### 3.3 *CLE5* and *CLE6* transcription is regulated by plant hormones

**Figure 5:**
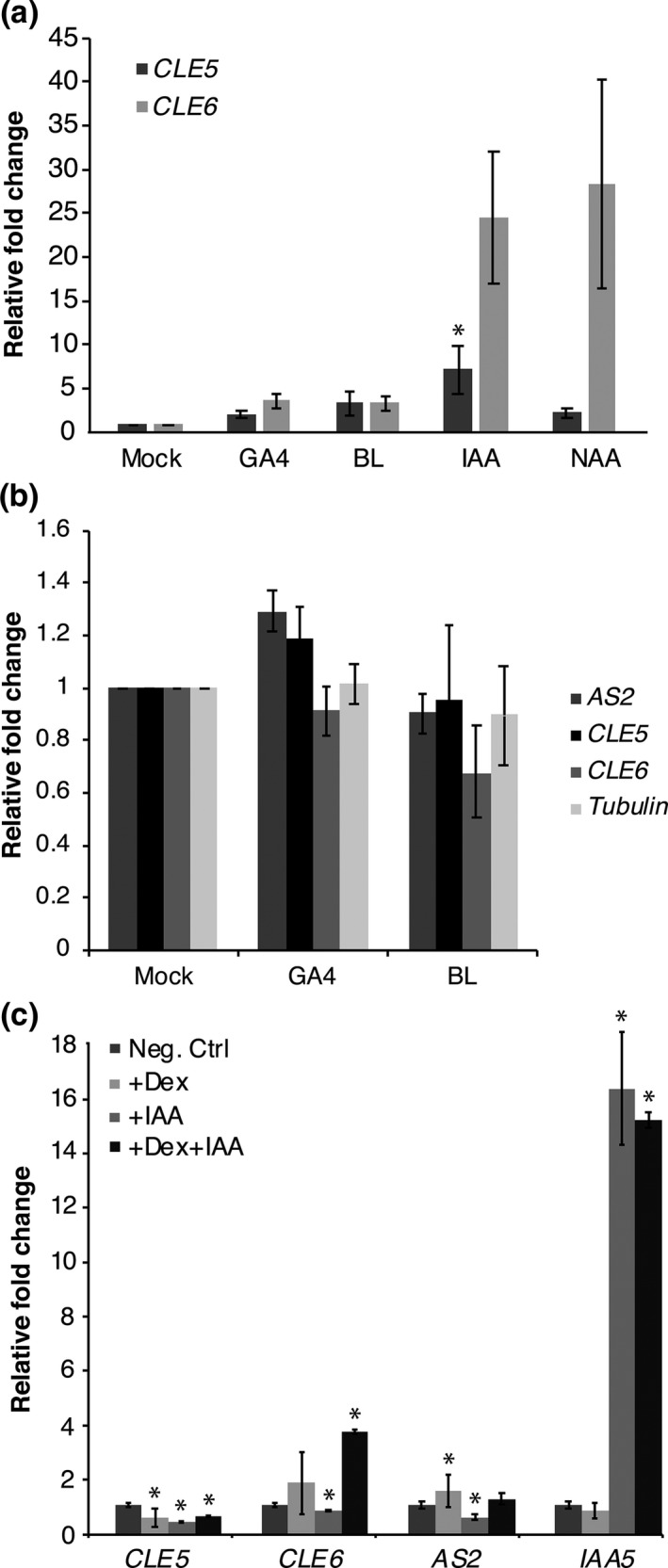
*CLE5* and *CLE6* expression in response to BOP1 induction in the absence or presence of hormones. (a) Relative fold change in *CLE5* and *CLE6* transcript levels in 11‐day‐old Col‐0 seedlings treated with gibberellin (GA_4_), brassinolide (BL), or auxin (IAA or NAA) for 4 hr. (b) Relative fold change in *AS2, CLE5* and *CLE6* transcript levels 11‐day‐old p35S:*BOP1‐GR bop1‐1* seedlings treated with Dex plus either GA_4_ or BL for 4 hr. (c) Relative fold change in *CLE5, CLE6, AS2,* and *IAA5* transcript levels of 11‐day old p35S:*BOP1‐GR bop1‐1* seedlings treated with Dex and/or IAA for 4 hr. Expression values (mean ± *SD*) were normalized to *MON1* and asterisks indicate a significant difference from the wild‐type mean at *p* < 0.05

Because hormones have long been implicated in regulation of leaf development and architecture, we sought to define the effect of various hormones on *CLE5* and *CLE6* expression in developing Arabidopsis rosette leaves. First, we treated 11‐day‐old wild‐type seedlings with 30 μM gibberellin (GA_4_), 2 μM brassinolide (BL), or 10 μM indole‐3‐acetic acid (IAA), a naturally occurring auxin, for 4 hr and Col‐0 quantified *CLE5* and *CLE6* transcription levels using RT‐qPCR. We found that the mRNA levels of both genes were slightly elevated in after application of both GA_4_ and BL (Figure 5a), indicating that the two *CLE* genes respond to hormones that regulate leaf formation. Interestingly, IAA treatment led to a much higher upregulation of *CLE5* and *CLE6* expression (Figure 5a), with *CLE6* transcription showing a ~24‐fold induction. Thus both the *CLE5* and especially the *CLE6* gene appear to be auxin responsive.

Next we examined whether phytohormones played a role in BOP1‐mediated regulation of *CLE5* and *CLE6* expression. We treated 11‐day‐old p35S:BOP1‐GR *bop1‐1* seedlings with 10 μM Dex alone or together with 20 μM GA_4_, 2 μM BL, or 10 μM IAA for 4 hr and quantified *CLE5* and *CLE6* transcription levels using RT‐qPCR. No significant change in *CLE5* or *CLE6* mRNA expression levels was observed following application of both +Dex and +GA, or of both +Dex and +BL (Figure 5b). This indicates that neither GA nor BL affects the BOP1‐mediated regulation of *CLE5* or *CLE6* transcription, nor vice versa. In contrast, the application of both +Dex and +IAA resulted in differential effects on *CLE5* and *CLE6* expression. For *CLE5*, BOP1‐GR induction by 4 hr of Dex treatment led to a moderate reduction in *CLE5* transcript levels, as did 4 hr application of 10 μM IAA (Figure 5c). Simultaneous treatment with both Dex and IAA for 4 hr did not further reduce *CLE5* mRNA levels, indicating that the two treatments did not have cumulative effects. Our data indicate that in a *bop1‐1* background *CLE5* is repressed by Dex application, as shown earlier (Figure 3b), as well as by exogenous auxin. In the case of *CLE6*, BOP1‐GR induction by Dex treatment alone led to no significant change in *CLE6* transcript levels, whereas IAA treatment resulted in a slight reduction in *CLE6* mRNA levels (Figure 5c). *CLE6* transcript levels were much more mildly affected by IAA treatment in *bop1‐1* plants (Figure 5c) than in Col plants (Figure 5a). Upon simultaneous treatment with both Dex and IAA for 4 hr, *CLE6* transcription was elevated beyond the levels detected upon BOP1‐GR induction alone (Figure 5c). These results suggest that *CLE6* transcript levels are independently regulated by BOP1 and auxin. Thus the *CLE5* and *CLE6* genes show differential regulation by BOP1 and phytohormones in developing leaves.

### 3.4 *CLE5* and *CLE6* have mild effects on overall leaf shape

In order to determine the function of *CLE5* and *CLE6* during Arabidopsis development we generated null alleles of the two genes using the CRISPR/Cas9 genome engineering technology (Feng et al., 2013; Nekrasov, Staskawicz, Weigel, Jones, & Kamoun, 2013). Transformation of wild‐type Col‐0 plants with a single guide RNA (sgRNA) construct targeted to the *CLE5* or *CLE6* coding sequences yielded multiple independent transformants. Independent T1 plants were self‐fertilized and homozygous individuals were identified in the T2 or T3 generations by restriction enzyme digestion and sequencing.

**Figure 6:**
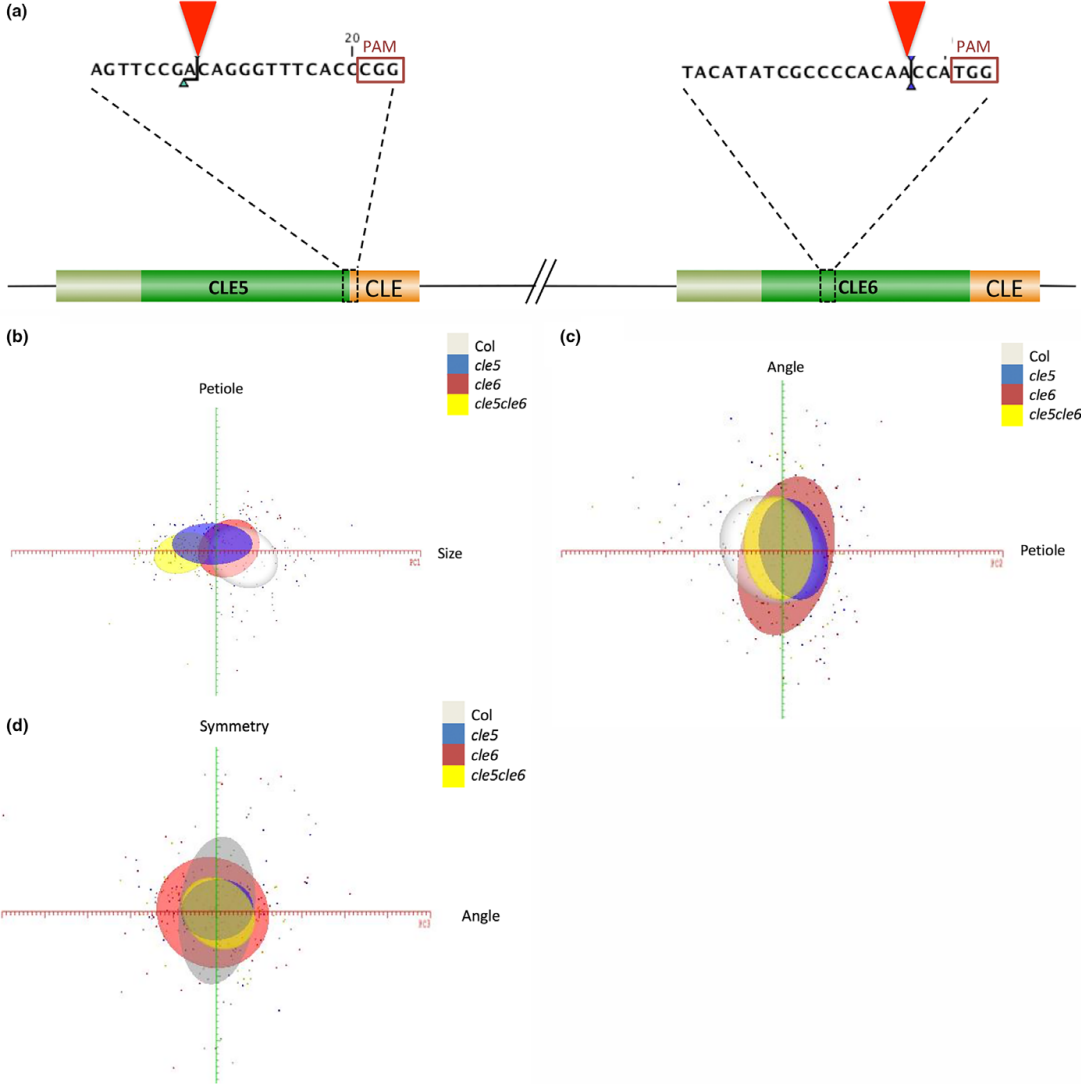
*CLE5* and *CLE6* loss‐of‐function allele generation and role in leaf formation. (a) Locations of the *cle5* and *cle6* CRISPR‐Cas9 induced mutations (red arrowheads) upstream of the PAM site (red box) within the sgRNA for the *CLE5* and *CLE6* coding sequences. The coding sequences of the signal peptides are represented in olive, the variable domains in green, and the CLE domains in orange. (b–d) Two‐dimensional PC maps generated using ≥5 standard deviations from the mean leaf along the *X*‐axis PC (red) and ≥2.5 standard deviations along the *Y*‐axis PC (green). Each standard deviation is represented by a major tick on the axis, and each leaf measured is represented by a single colored point. (b) Variation along PC1 and PC2 for Col‐0 (white oval), *cle5* (blue oval), *cle6* (red oval), and *cle5 cle6* (yellow oval) leaves. (c) Variation along PC2 and PC3 for each genotype. (d) Variation along PC3 and PC4 for each genotype

Two independent *CLE5* and two independent *CLE6* homozygous lines were chosen for further study (Figure 6a). One *cle5* allele consisted of an insertion of an “A” nucleotide at position +215 downstream of the translation start site, and was designated *cle5‐1*. A second allele contained a deletion of a five nucleotides starting at position +210 and was designated *cle5‐2*. The *cle5‐1* mutation introduces a frameshift that alters the amino acid sequence of the CLE domain beginning at the third residue, while the *cle5‐2* mutation deletes the first three residues of the CLE domain and also introduces a frameshift. The first *cle6* allele consisted of an insertion of an “A” nucleotide at position +106 downstream of the translation start site, and was designated *cle6‐1*. A second allele contained a deletion of four nucleotides starting at position +102 and was designated *cle6‐2*. Each of these mutations generates a frameshift in the *CLE6* coding sequence upstream of the CLE domain. Due to the nature of the mutations none of these *cle5* or *cle6* alleles produces a functional CLE polypeptide, and thus they represent loss‐of‐function alleles.

Because *CLE5* and *CLE6* loci are located approximately 1.7 kb apart in a head‐to‐tail arrangement on chromosome 2, their proximity makes it improbable to produce double mutants by conventional genetic crosses. Therefore we took advantage of genome engineering to target both *CLE5* and *CLE6* for simultaneous mutation by transforming wild‐type Col‐0 plants with a construct that contains both of the sgRNAs used in the previous experiments, the one targeted to the *CLE5* coding sequence and the one targeted to the *CLE6* coding sequence. Three doubly homozygous mutants were obtained, one of which contained an insertion of an “A” nucleotide at position +215 downstream of the *CLE5* translation start site as well as an insertion of an “A” nucleotide at position +106 downstream of the *CLE6* translation start site. Because each mutation generates a frameshift in the respective *CLE5* or *CLE6* coding sequence upstream of the CLE domain, no functional polypeptides are generated and this *cle5‐3 cle6‐3* double mutant represents a knockout of both genes.

We next performed a large‐scale morphological analysis of Col‐0, *cle5‐1, cle6‐1,* and *cle5‐3 cle6‐3* plants from germination through the vegetative phase of development. We observed no significant differences in leaf initiation rate, total rosette leaf number, mature rosette leaf size, rosette diameter, or flowering time under different photoperiods between Col‐0 and *cle5, cle6,* or *cle5 cle6* plants. We also analyzed the size, number, composition, and morphology of wild‐type and mutant rosette leaf cells using scanning electron microscopy (SEM) and histological analysis but detected no differences between the genotypes. WOX1 and PRS promote leaf margin cell file formation (Nakata et al., 2012), with *prs wox1* rosette leaves having fewer and p35S:*PRS* leaves having more margin cell files than wild‐type rosette leaves (Supporting Information Figure S2). Because *wox1‐1* and *prs‐1* plants display reduced *CLE5* and *CLE6* expression, we used SEM to assess whether mutations in *CLE5* and/or *CLE6* caused leaf margin cell file defects. However, we found no reproducible differences in margin cell file number, morphology or fate between Col‐0, *cle5, cle6,* and *cle5 cle6* rosette leaves (Supporting Information Figure S2). Thus *CLE5* and *CLE6* separately or together have no macroscopic effects on vegetative development under normal growth conditions.

Finally, we conducted a leaf morphometric study to identify any subtle phenotypes attributable to the loss of *CLE5* or *CLE6* activity. The LeafAnalyser image processing program (Weight et al., 2008) was used to quantify the shape of Col‐0, *cle5‐1, cle6‐1,* and *cle5‐3 cle6‐3* first through fourth rosette leaves by performing a principal component (PC) analysis of distinct aspects of overall rosette leaf shape among the different genotypes. LeafAnalyser parsed the major sources of variation in leaf shape into four principal components—leaf size, width, petiole angle, and tip‐to‐base asymmetry—that together account for 95% of the total Arabidopsis leaf shape variation. Using the LeafAnalyser software we measured each of the four PCs for at least 50 leaves per genotype, and then generated two‐dimensional PC maps by plotting the values for each genotype for two of the PCs (leaf size versus width, width versus petiole angle, etc.). Each oval represented one standard deviation from the mean leaf for one genotype, such that the extent of overlap between ovals showed the relative similarity in phenotype.

This morphological analysis revealed modest effects of *CLE5* and *CLE6* on leaf shape (Figure 6b–d). The first component, which accounted for almost 66% of the variation, was overall leaf size and was likely due to leaf age, independent of genotype. The second component was variation in leaf width that accounted for 12% of the variability and was most apparent in the *cle5* and *cle5 cle6* genotypes (Figure 6b). The third component was leaf curvature due to petiole angle and accounted for almost 10% of the total leaf shape variability. This variance was most evident in *cle6* leaves (Figure 6c). The fourth component, leaf symmetry, accounted for 7% of the variability and was detected in both single and double mutants (Figure 6d). While these effects are subtle, they do distinguish possible distinct roles for *CLE5* and *CLE6* in regulating leaf shape during development.

## 4 DISCUSSION

Vegetative development is a highly coordinated series of events that starts with a primordium initiated from the shoot apical meristem. This primordium undergoes pattern formation along three polarized axes, the establishment of the basic cell types of the petiole, lamina, and marginal structures, and a maturation process that involves cell differentiation and expansion to achieve the mature leaf shape (Bar & Ori, 2014). Long‐range hormonal signals such as auxin, GA, and BR are well‐characterized players in leaf development; however, their relative ubiquity and omnipresence due to diffusion and active transport likely limit their ability to regulate the highly coordinated and precise events required in organ development alone. While small peptides are known integral signals in plant root and vasculature cell development, we pose a model of CLE signaling that plays a role in regulating shoot organ formation in plants.

Analyses of *CLE* gene expression reveal *CLE5* and *CLE6* promoter activity in the aerial tissues of wild‐type Arabidopsis seedlings was uniquely confined to the area around the shoot apex, the base of the lateral organs, and the leaf hydathodes (Jun, Fiume, et al., 2010). We investigated the expression of *CLE5* and *CLE6* at higher resolution in aerial tissues of wild‐type Arabidopsis plants using promoter:*GUS* and in situ hybridization (Figure 1). Interestingly, the two closely related genes displayed distinct expression patterns. *CLE6* was expressed exclusively within the adaxial domain of developing rosette leaf primordia, while *CLE5* transcription was restricted to the lateral regions of the primordia but was detected in both the adaxial and abaxial domains. Thus, the upper, outer periphery of the developing leaves express both *CLE5* and *CLE6*, whereas the underside of the leaf margins expresses only *CLE5* and the midvein region expresses only *CLE6*. Our data show subtle but distinct differences between the *CLE5* and *CLE6* expression patterns despite the high degree of similarity between the two transcription units. The *CLE5* and *CLE6* genes encode precursor proteins with 77% identity and 84% similarity (Sharma, Ramirez, & Fletcher, 2003), and produce predicted mature CLE peptides with identical amino acid sequences (Cock & McCormick, 2001). The two genes, along with *CLE4* and *CLE7*, lie within a 16.5 kb region on chromosome 2. Within this gene cluster, the *CLE5, CLE6,* and *CLE7* coding regions are more similar to one another than to other members of the family, suggesting they may have arisen by local gene duplication events (Cock & McCormick, 2001). Nonetheless, analysis of 1.7 kb of upstream promoter sequence, corresponding to the distance between the *CLE5* stop codon and the *CLE6* start codon, between these three genes shows only 49.6% similarity between the *CLE5* and *CLE6* promoters. This is comparable to the 45.2% similarity between the *CLE5* and *CLE7* promoters, despite *CLE7* expression being restricted to the root (Jun, Fiume, et al., 2010). Therefore the divergence of the upstream regulatory regions between the *CLE5* and *CLE6* genes may account for the differences in their expression patterns. Still, determining how highly similar peptides are differentially regulated in space and time is crucial to understanding the roles of such signaling molecules in the larger context of plant development.

Our study reveals that *CLE5* and *CLE6* are downstream targets of two transcriptional regulators that affect leaf patterning, BOP1/2 and AS2. Both *CLE5* and *CLE6* are downregulated in *bop1 bop2* seedlings, showing that BOP1 and BOP2 are positive regulators of *CLE5* and *CLE6* transcription (Figure 2). Conversely, an increase in *CLE5/6* transcript levels in *as2‐1* seedlings indicates that AS2 negatively regulates *CLE5* and *CLE6* transcription. *AS2* transcripts are present throughout the adaxial leaf domain (Byrne et al., 2000; Iwakawa et al., 2007), whereas *BOP1* and *BOP2* expression is confined to the most proximal end of the adaxial domain (Ha et al., 2004; Norberg et al., 2005). This suggested that BOP1/2 might induce *CLE5/6* transcription at the base of the petiole and AS2 might repress it in more distal positions along the petiole and in the blade. However, in situ hybridization experiments indicated that the absence of neither BOP1/2 nor AS2 altered the *CLE6* expression domain (Figure 2), indicating that BOP1/2 and AS2 affect *CLE5* and *CLE6* transcript levels within their native domain rather than establishing the boundaries of their spatial expression domains.

Additional experiments showed that *CLE5* and *CLE6* are differentially regulated by BOP1/2 and AS2. *CLE6* transcription was upregulated in shoot tissues within 1 hr in response to BOP1‐GR translocation (Figure 3), suggesting that *CLE6,* like *AS2*, might be a direct target of BOP1 activation. However, using ChIP‐qPCR we detected no BOP1 binding to the *CLE6* (or the *CLE5)* regulatory region (Supporting Information Figure S1). Thus either the regulation of *CLE6* transcription by the BOP proteins is indirect or additional rate‐limiting factors are required for BOP1 binding to the *CLE6* locus. Our data also show that the inductive effect of BOP1 on *CLE6* transcription overcomes the repressive effect of AS2 we observed in the *as2‐1* background (Figure 2). This oppositional effect of AS2 and BOP1/2 on *CLE6* expression indicates that a combination of activators and repressors likely controls *CLE* gene transcription in developing leaves.

In contrast to *CLE6, CLE5* expression was not upregulated upon BOP1‐GR translocation (Figure 3), which indicates that BOP1 is sufficient to induce *CLE6* but not *CLE5* transcription in leaves. Based on control experiments (Figure 3), the slight reduction observed in *CLE5* transcript levels is more likely to be a result of Dex application itself, as has been noted elsewhere (Kang, Fang, & Singh, 1999), than of targeted transcriptional regulation by BOP1. Finally, we detected no significant affect on either *CLE5* or *CLE6* transcription in the AS2‐GR system (Figure 3). One possible explanation is that over‐expression of AS2 alters the expression or activity of its partner AS1. Alternatively, the amount of endogenous AS1 may be a rate‐limiting factor for the activity of the complex, rendering the excess AS2 protein unable to affect target gene transcription.

WOX transcription factors are known key components of CLE signaling pathways, and *WOX* transcription factor genes have been linked to the promotion of leaf blade outgrowth in several plant species. These include *LAM1* in *Nicotiana sylvestris* (Lin et al., 2013), *STF* in *Medicago truncatula* (Tadege et al., 2011), *narrow sheath1* and *2* in maize (Nardmann, Ji, Werr, & Scanlon, 2004), and *narrow leaf2* and *3* in rice (Ishiwata et al., 2013). In Arabidopsis, the closely related *WOX1* and *PRS* (aka *WOX3*) genes have described roles in leaf development (Nakata et al., 2012), during which their expression is induced by auxin (Caggiano et al., 2017). *prs wox1* leaves display reduced blade outgrowth and fewer margin cell files (Nakata et al., 2012) while *PRS* over‐expressor lines show an increase in leaf margin cell file number (Supporting Information Figure S2), indicating that *PRS* and *WOX1* promote blade outgrowth and margin cell file formation. Our expression analysis reveals that both WOX1 and PRS are positive regulators of *CLE5* and *CLE6* transcription (Figure 4). This is consistent with a role for a WOX‐CLE signaling module in leaf development and provides further evidence that multiple factors regulate *CLE* gene expression and organize leaf development. Based on the subtle leaf shape defects in *cle5 cle6* seedlings we propose that *CLE5* and *CLE6* may act downstream of *PRS* and *WOX1* in regulating lamina outgrowth. In contrast, no obvious leaf margin cell file phenotype was observed in *cle5* or *cle6* single or double mutant plants (Supporting Information Figure S2), either because *CLE5* and *CLE6* are not required for margin cell development or because their loss is compensated by other *CLE* genes.

In addition to transcriptional regulators, several long‐range hormones have well‐established roles in plant organ development. GA acts subsequent to initial patterning events to regulate the rate of cell proliferation and expansion during leaf outgrowth (Achard et al., 2009). In addition, auxin, ABA and BL all play roles in regulating the transition from leaf growth by cell division to growth by cell expansion (Kalve et al., 2014). GA is known to promote *CLE6* transcription in the root stele, and ectopic expression of *CLE6* has been shown to partially compensate for GA‐deficiency during vegetative growth as well (Bidadi et al., 2014). Our work shows that *CLE5* and *CLE6* are indeed responsive to GA in shoot tissues, as well as to BL and IAA (Figure 5). *CLE5* and *CLE6* expression is slightly induced by GA (Figure 5), which promotes leaf differentiation, and thus the *CLE* genes may act downstream of GA (and/or other hormones) during the later stages of leaf differentiation to achieve the mature leaf morphology.

The transcription of both *CLE5* and *CLE6* is also auxin responsive, with *CLE6* responding more strongly to IAA application than *CLE5* (Figure 5). Interestingly, simultaneous BOP1‐GR induction and auxin application resulted in differential effects on *CLE5* and *CLE6* transcription. Whereas *CLE5* mRNA expression levels were slightly reduced, *CLE6* transcription levels were elevated in the presence of both BOP1 and auxin (Figure 5). These data reveal that *CLE5* and *CLE6* undergo differential regulation by BOP1 and phytohormones, again indicating that a combination of activators and repressors are required to control *CLE* gene expression in developing leaves.

Although gross morphological phenotypes were not visible in *cle5/6* single or double mutant plants, more sensitive morphometric analysis showed that the two genes affect the final shape of the rosette leaves. Specifically, variation in leaf petiole width (PC2) accounted for 12% of the variability in leaf shape and was most apparent in the *cle5* and *cle5 cle6* genotypes (Figure 6). This subtle effect on petiole width is reminiscent of the blade‐on‐petiole phenotype of *bop* leaves, where formation of ectopic blade tissue along the petiole results in a wider than normal leaf base (Ha et al., 2003, 2007). Thus, the regulation of *CLE5* and *CLE6* expression by the BOP proteins appears to be important for fine‐tuning leaf formation by limiting the lateral growth of the petiole. Collectively, our data are consistent with a scenario in which BOP1/2, AS2, PRS/WOX1, and phytohormones act combinatorially to modulate *CLE5* and *CLE6* transcription at the leaf base to levels appropriate to produce the final leaf shape, although additional experiments will be necessary to fully clarify the relationships between these various factors. A role for *CLE5* and *CLE6* in regulating the activity of differentiating cells during the later stages of leaf development contrasts with that of most other *CLE* genes functionally characterized to date, which predominantly affect undifferentiated, meristematic cells (Fletcher, Brand, Running, Simon, & Meyerowitz, 1999; Gutierrez‐Alanis et al., 2017; Hirakawa et al., 2008; Stahl et al., 2009; Whitford et al., 2008).

The fact that two genes that encode identical CLE peptides are expressed in overlapping but not identical patterns and are differentially regulated by upstream hormones and transcription factors may be an evolutionary artifact or may have functional significance. To date we have not observed a unique function for either *CLE5* or *CLE6* in organ development. However, overlapping domains of genes expressing secreted peptides may contribute to dose‐dependent signaling events that functional analysis at the level of single cells may be required to uncover. Such multiple independent yet cross‐talking regulatory mechanisms may provide the range of distinct signaling events required for the highly coordinated development of an organ with three polar axes and multiple specific cell types.

## ACCESSION NUMBERS

AS2 (At1g65620), BOP1 (At3g57130), BOP2 (At2g41370), CLE5 (At2g31083), CLE6 (At2g31085), MON1 (At2g28390), PRS (At2g28610), TUB2 (At5g62690), WOX1 (At3g18010).

## AUTHOR CONTRIBUTIONS

PD, EG, JHJ and JCF designed the research; PD, EG, TD and JHJ performed the research; EG contributed new tools; PD, EG, JHJ, TD and JCF analyzed the data; and PD and JCF wrote the paper with input from the other authors.
