# Supplementary figures and images for: Peptide signaling molecules CLE5 and CLE6 affect Arabidopsis leaf shape downstream of leaf patterning transcription factors and auxin
Source: Plant Direct. 2018 Dec 20;2(12):e00103. doi: 10.1002/pld3.103 (PMC6508849; doi:10.1002/pld3.103)

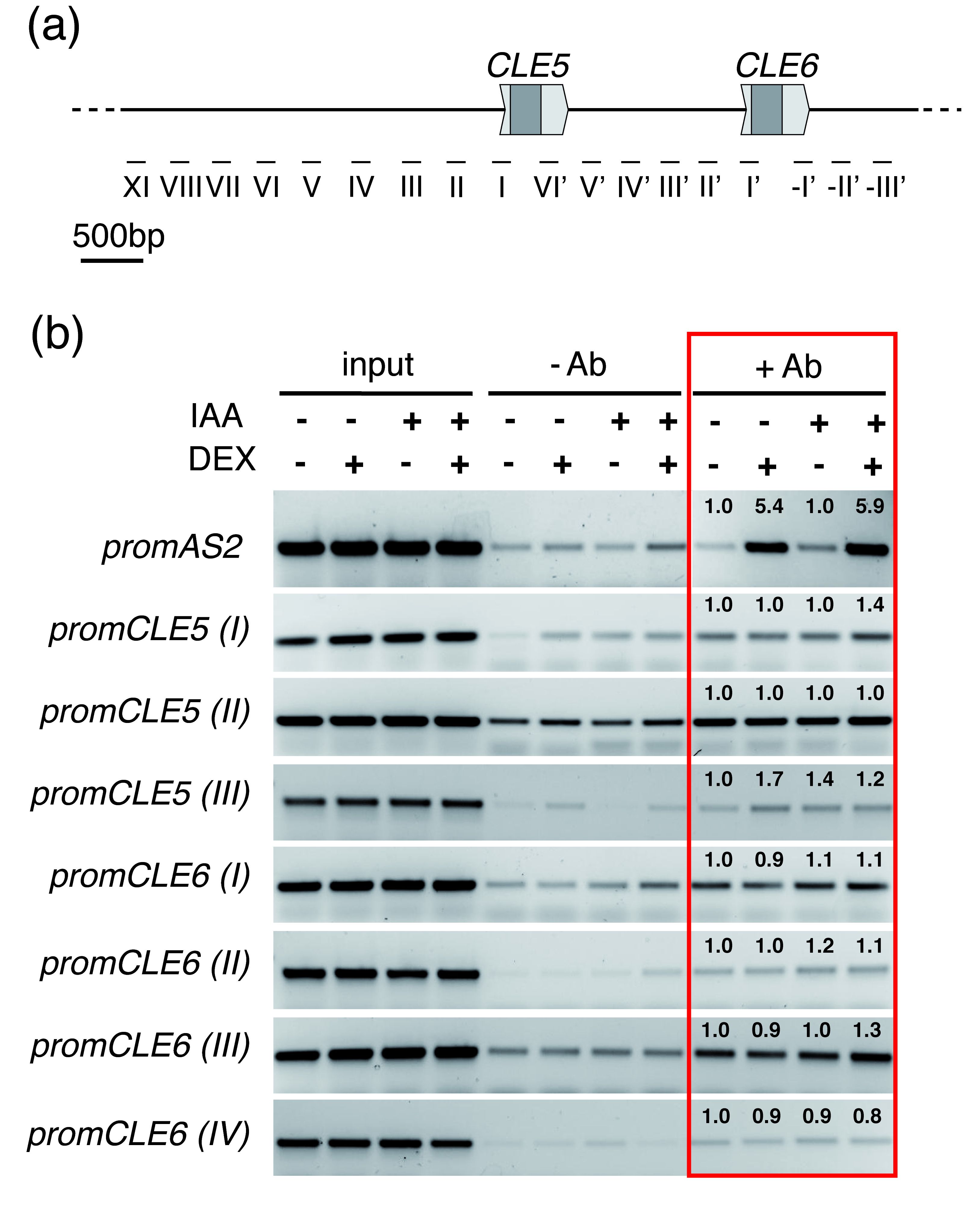

Supplement: Supplementary file 1 [file PLD3-2-e00103-s001.tif]

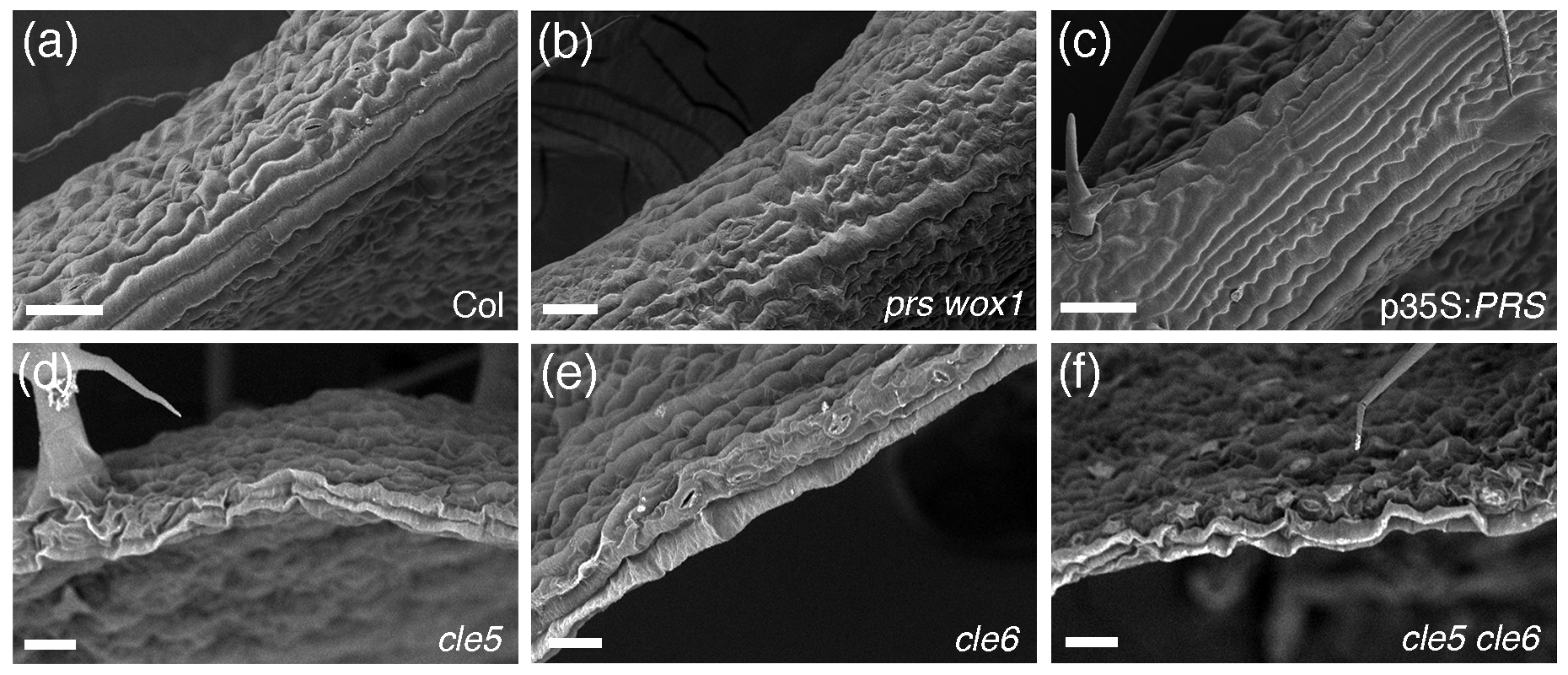

Supplement: Supplementary file 2 [file PLD3-2-e00103-s002.tif]
