# Supplementary material for: Peptide signaling molecules CLE5 and CLE6 affect Arabidopsis leaf shape downstream of leaf patterning transcription factors and auxin
Source: Plant Direct. 2018 Dec 20;2(12):e00103. doi: 10.1002/pld3.103 (PMC6508849; doi:10.1002/pld3.103)
Supplement: Supplementary file 3 [file PLD3-2-e00103-s003.docx]

**Table S1. Primer sequences used in the study.**

**Name Sequence**

Cloning

pBOP1(-5797) FW 5’- TGGGAAGCGGAGTGTAATTC

pBOP1 RV 5’- CATAGCTCCTTTGTTGATTTCTTTGATAC

pBOP1:CLE6 FW 5’- CAAAGAAATCAACAAAGGAGCTATGGCGAATTTGATCCTTAAGC

CLE6_CDS+Stop RV 5’- TCAATGGTGTTGTGGATCAG

T7_promoter 5’- TAATACGACTCACTATAG

pCR8_FW 5’- GTTTTCCCAGTCACGACGTT

CLE5 P1 5’- GATTGACACGGCACGATATGAACGG

CLE5 P2 5’- AAACCCGTTCATATCGTGCCGTGTC

CLE6 P1 5’- GATTGCCCCACAACCATGGGCGATA

CLE6 P2 5’- AAACTATCGCCCATGGTTGTGGGGC

RT-qPCR

CLE5 FW 5’- GCTCGAATCCTCCGTTCATA

CLE5 RV 5’- TCGCCTCTCGTTATGACCTT

CLE6 FW 5’- CGTGAACTCGGGATTGATCT

CLE6 RV 5’- CAATGGTGTTGTGGATCAGG

PRS FW 5’- ACGGAGAATGAGTCCTGTGG

PRS RV 5’- TGTTGTATCTGCACCGCATT

WOX1 FW 5’- TTCCTCCAACATGTCCAACA

WOX1 RV 5’- TGTGTTCGTTGCTTCTCTCG

IAA5 FW 5’- TCACCGAACTACGGCTAGGT

IAA5 RV 5’- ACACATTCACTTTCCTTCAACG

MON1 FW 5’- AACTCTATGCAGCATTTGATCCACT

MON1 RV 5’- TGATTGCATATCTTTATCGCCATC

AtBT (tubulin) FW 5’- TGGGAACTCTGCTCATATCT

AtBT (tubulin) RV 5’- GAAAGGAATGAGGTTCACTG

ChIP-qPCR

pAS2 FW 5’- AATGCAATATGCAACCACGA

pAS2 RV 5’- CTAACGAAGAAGCGTGCAAA

pCLE5 I FW 5’- CCCACTCATGACCCAAACTT

pCLE5 I RV 5’- GGGTGGGAGTTGAGAGAGAG

pCLE5 II FW 5’- TCCTAGTGAACCTTATGTCAAGCA

pCLE5 II RV 5’- AACGCTCGGAATTCTCATCA

pCLE5 III FW 5’- CAAACCTGAAAATCATCAGAATG

pCLE5 III RV 5’- TGGACGAAAACAATTGTACCTG

pCLE5 IV FW 5’- GAGCTTTCCAAAGCCAAAAA

pCLE5 IV RV 5’- CGGTAAAATGTTTTGGTCCAT

pCLE5 V FW 5’- CGGTCCCCTAAAACATAATACG

pCLE5 V RV 5’- TGTGTTTCTTTTTGTAGAAACATTG

pCLE5 VI FW 5’- TGCTTGGTTTATTTGGTTTCA

pCLE5 VI RV 5’- TGTTGCAAATGCCACTTTCT

pCLE5 VII FW 5’- AAGCAGAAGAATGGTTTGACA

pCLE5 VII RV 5’- GGGAGACGAAGAAGCAGTTG

pCLE5 VIII FW 5’- ATTCGCAGTTGAATCGCATA

pCLE5 VIII RV 5’- AATAGTTCAGCGCGTCATCA

pCLE5 IX FW 5’- TGTGGGCATGTTTTCATTTC

pCLE5 IX RV 5’- CTCAACGGCGAGAAACAAAT

pCLE5 X FW 5’- CAACCGAATAAAAGTGAGAATGG

pCLE5 X RV 5’- TGCCACTCAACAACCACATT

pCLE5 XI FW 5’- CCGACGACGACTAACTGTCA

pCLE5 XI RV 5’- TGCTCTTTGATTCGGTTTGA

pCLE6 I’ FW 5’- TCCTTAACCTGTTCCCGTTTT

pCLE6 I’ RV 5’- TCGCCATTAAAGGTGATTAAGAA

pCLE6 II’ FW 5’- TTTCGATCGTTAAGGGTCAACT

pCLE6 II’ RV 5’- GCCGAATCCTACGCATATTT

pCLE6 III’ FW 5’- ACCGCAAAAGAAATCCATGA

pCLE6 III’ RV 5’- TCGAAATTCTCAGGTGGAAA

pCLE6 IV’ FW 5’- TTGATCGGCTATCCTCAGAA

pCLE6 IV’ RV 5’- GCAATGGAGTCATCTTTGTAGG

pCLE6 V’ FW 5’- GATTGGACGTCTTAGCACTTCA

pCLE6 V’ RV 5’- TTCGAATTAACCCTCTAAAACCTC

pCLE6 VI’ FW 5’- CCGAAGATCCAAGCACAAAT

pCLE6 VI’ RV 5’- TGTGGTACAAGGATACCAAAACC

pCLE6 –I’ FW 5’- AGTGGATTCCGAAAGGGTTT

pCLE6 –I’ RV 5’- TGACTTGCATGGATCAGTCAC

pCLE6 –II’ FW 5’- TTGGCTATTTCCCCTGTCTTT

pCLE6 –II’ RV 5’- ATCAGCTGAAAAGCATGCAA

pCLE6 –III’ FW 5’- TCTGCCACCAGTTGAAAAGA

pCLE6 –III’ RV 5’- TCCTGTTGCCATGAAAAGAA

Genotyping

CLE5CR_FW 5’- AACGATTAAAACCGGGGAAC

CLE5CR_RV 5’- AACGATTAAAACCGGGGAAC

CLE6CR_FW 5’- TTTCGATCGTTAAGGGTCAACT

CLE6CR_RV 5’- GCCAATCGCTGTTACAAAAA

sgRNA_FW 5’- AGAAGAGAAGCAGGCCCATT

sgRNA_RV 5’- TTCCCAAGGTCCAAAGACAC
