# Supplementary material for: Peptide signaling molecules CLE5 and CLE6 affect Arabidopsis leaf shape downstream of leaf patterning transcription factors and auxin
Source: Plant Direct. 2018 Dec 20;2(12):e00103. doi: 10.1002/pld3.103 (PMC6508849; doi:10.1002/pld3.103)
Supplement: Supplementary file 4 [file PLD3-2-e00103-s004.pdf]

Dear Dr. Forbes,

Below is our response to the final reviewer suggestion, highlighted in red text:

• My single concern is the following item:" ...To conclude, the data regarding BOP1, AS2, WOX1- mediated CLE5/CLE6 expression is not strong enough at this moment. The same regarding the auxin treatment. Whereas in Fig.5 a the authors show x24 increase in CLE6, in Fig.5c after similar treatment the CLE6 expression was only slightly changed. These data is a little bit confusing"
Therefore, I suggest that the authors rewrite the sentences related to these aspects highlighting the different genetic background used in some experiments and mentioning that though the results indicate that CLE5 and CLE6 affect leaf shape downstream of leaf patterning transcription factors and auxin more studies would be needed to ascertain the statement.

We have added and revised several sentences to address this point, in the Results on page 14 and in the Discussion on page 22.
